# Supplementary material for: Donor-delivered cell wall hydrolases facilitate nanotube penetration into recipient bacteria
Source: Nat Commun. 2020 Apr 22;11:1938. doi: 10.1038/s41467-020-15605-1 (PMC7176660; doi:10.1038/s41467-020-15605-1)
Supplement: Supplementary file 1 — Supplementary information [file 41467_2020_15605_MOESM1_ESM.pdf]

**Supplementary Information**

# Donor-delivered cell wall hydrolases facilitate nanotube penetration into recipient bacteria

**Baidya et al.**

## Supplementary Figures

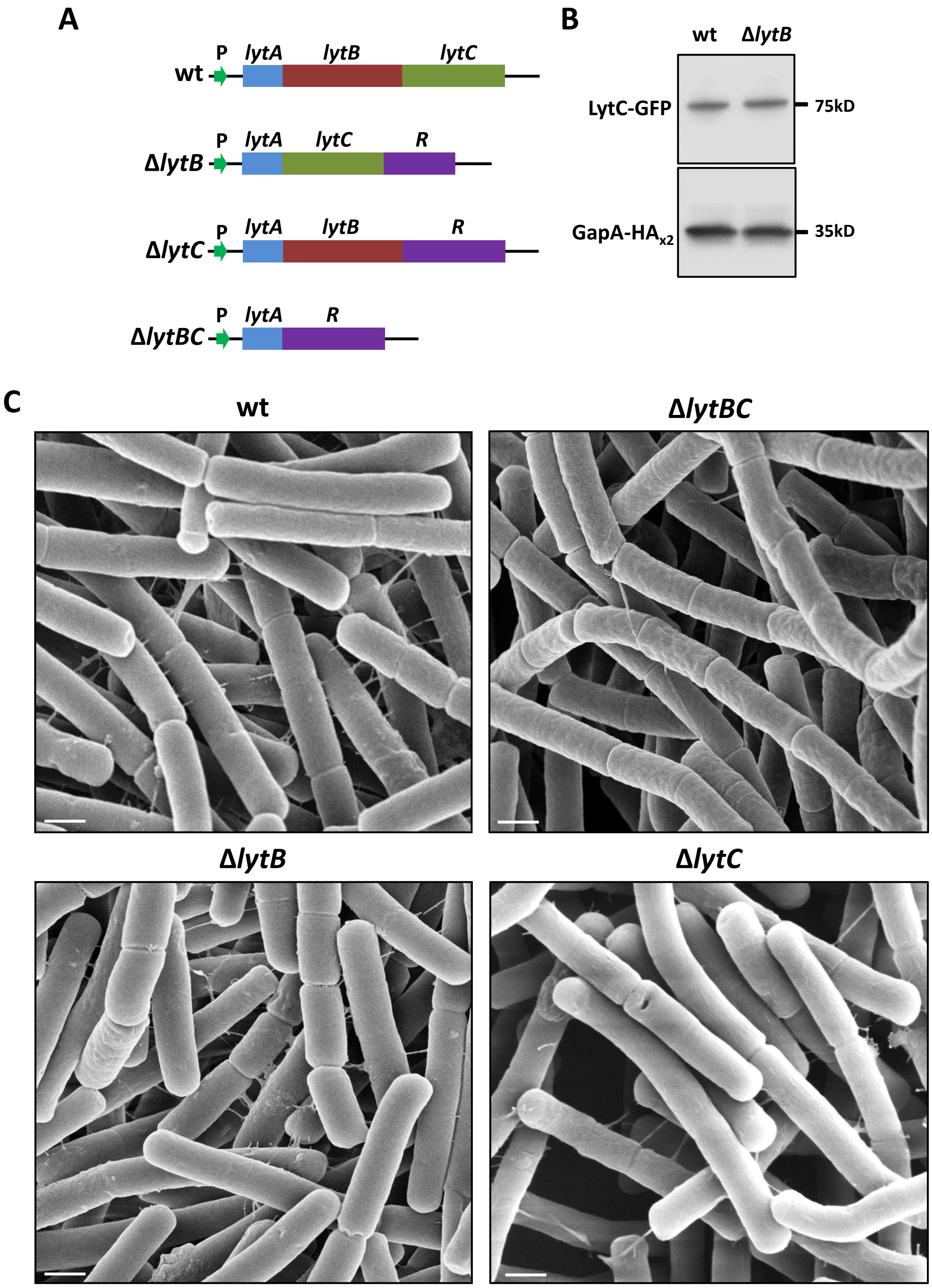

**Supplementary Figure 1: LytB and LytC impact nanotube formation**

(**A**) Schematic depiction of the *Bs lytABC* operon, based on *Subti*Wiki, and the derived Δ*lytB,* Δ*lytC* and Δ*lytBC* mutants. P represents the promoter region, and *R* indicates an antibiotic resistance gene.

(**B**) Total protein was extracted from equal number of *Bs* wt (AB281: *lytC-gfp*, *gapA-HA*$_{x2}$) and Δ*lytB* (AB281a: *lytC-gfp*, Δ*lytB*, *gapA-HA*$_{x2}$) cells and subjected to Western blot analysis utilizing anti-GFP antibodies to monitor the levels of LytC-GFP. GapA-HA$_{x2}$ levels, detected on a separate western blot with anti-HA antibodies, serve as a loading control. The results indicate that LytC production is unperturbed following deletion of *lytB*. Source data are provided as a Source Data file.

(**C**) *Bs* Δ*lytB* (AB232: Δ*lytB*, *Δhag*) and *ΔlytC* (AB62: *ΔlytC*, Δ*hag*) strains were grown to mid logarithmic phase, spotted onto EM grids, incubated on LB agar plates for 4 h at 37°C, and visualized by XHR-SEM. *Bs* wt and *Bs ΔlytBC* are large fields of cells that correspond to Figure 1A and 1B. Scale bar represents 500 nm.

The experiments were repeated at least 3 times independently with similar results.

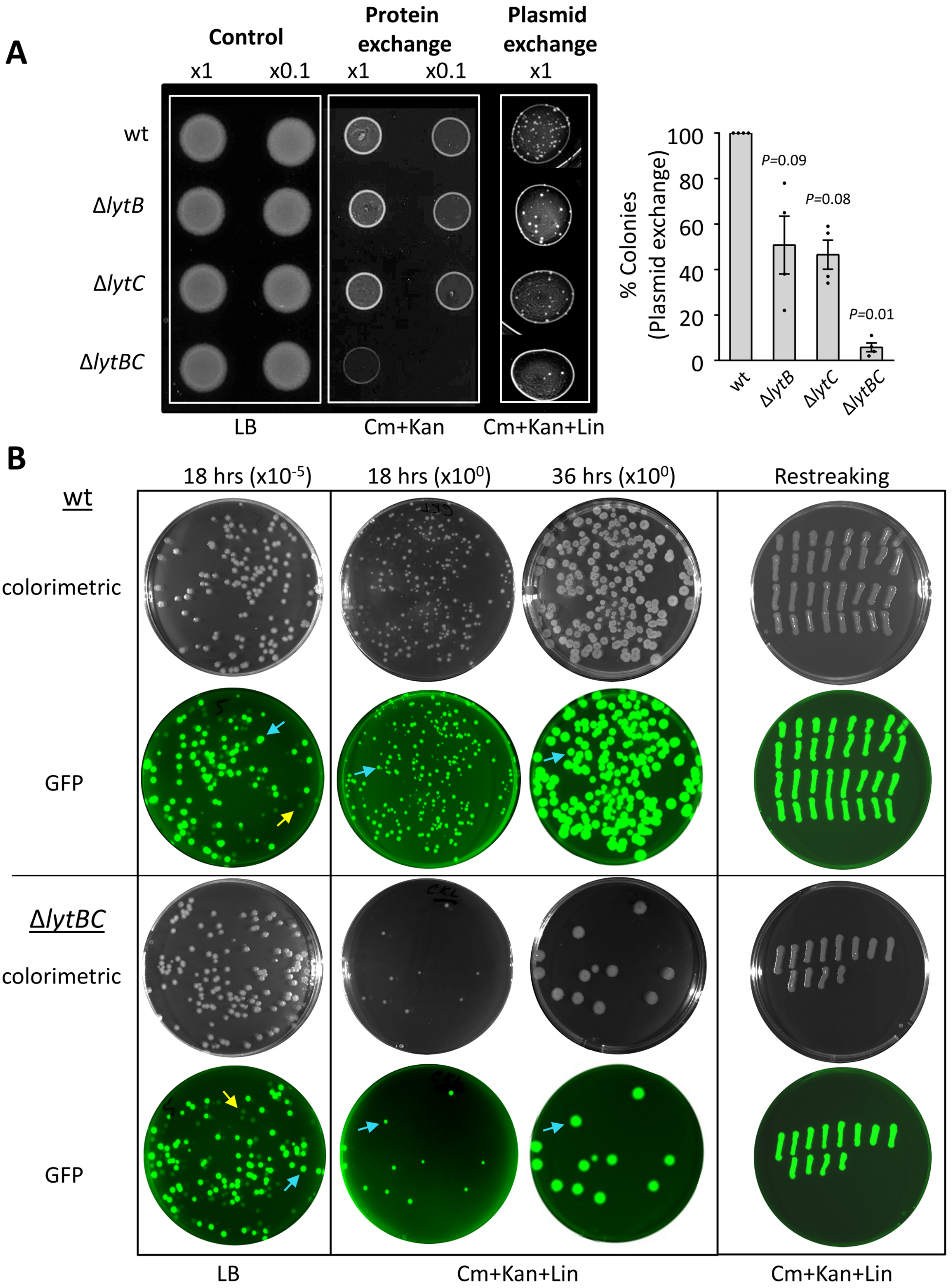
A
Control
x1
x0.1
Protein exchange
x1
x0.1
Plasmid exchange
x1
wt
ΔlytB
ΔlytC
ΔlytBC
LB
Cm+Kan
Cm+Kan+Lin
% Colonies (Plasmid exchange)
100
80
60
40
20
0
P=0.09
P=0.08
P=0.01
wt
ΔlytB
ΔlytC
ΔlytBC
B
18 hrs (x10-5)
18 hrs (x100)
36 hrs (x100)
Restreaking
wt
colorimetric
GFP
ΔlytBC
colorimetric
GFP
LB
Cm+Kan+Lin
Cm+Kan+Lin

**Supplementary Figure 2: LytB and LytC impact intercellular molecular exchange**

(**A**) Assessing molecular exchange in Δ*lytB*, Δ*lytC* and Δ*lytBC*. For protein exchange assay, pairs of a donor (SB463: *amyE*::$P_{hyper\text{-}spank}$-*cat-spec*) ($Cm^R$, $Spec^R$) and a recipient (SB513: *amyE*::$P_{hyper\text{-}spank}$-*gfp-kan*) ($Kan^R$) parental strains (wt) were used. The investigated mutants harbor the corresponding genotypes and carry the indicated null mutation in both donor and recipient strains. Donor and recipient strains were mixed in 1:1 ratio (at two concentrations x1, x0.1) and incubated in LB supplemented with 1 mM IPTG for 4 h at 37°C with gentle shaking. Equal numbers of cells were then spotted onto LB agar (Control) and LB agar containing chloramphenicol (Cm) and kanamycin (Kan) (Protein exchange). For plasmid exchange assay, pairs of a donor (GD110: *amyE*::$P_{hyper\text{-}spank}$-*cat-spec*, pHB201/*cat, erm*) ($Cm^R$, $Spec^R$, $Mls^R$) and a recipient (SB513: *amyE*::$P_{hyper\text{-}spank}$-*gfp-kan*) ($Kan^R$) parental strains (wt) were used. The investigated mutants harbor the corresponding genotypes and the indicated null mutation in both donor and recipient strains. Cells were mixed in 1:1 ratio (concentration x1), processed as described for protein exchange, and spotted onto LB agar containing Cm, Kan and lincomycin (Lin) (Plasmid exchange). The graph shows the relative number (wt = 100 %) of colonies obtained on Cm+Kan+Lin antibiotic plate, reflecting the frequency of plasmid exchange. Shown are mean ± SEM and *P* values (unpaired student's t-test) of at least 3 independent experiments. Source data are provided as a Source Data file.

(**B**) Extension of plasmid exchange analysis. Donor and recipient pairs of wt (upper panels) and Δ*lytBC* (lower panels) were mixed and processed as described in A for plasmid exchange. Equal amount of cells from wt and Δ*lytBC* were then plated on LB agar (left panels), and on LB agar containing Cm, Kan and Lin (middle panels) at the

indicated dilutions. Plates were imaged after 18 and 36 hrs of incubation by colorimetric imaging and by a fluorescent reader to indicate the presence of recipient cells harboring GFP. The obtained colonies were re-streaked over plates containing Cm, Kan and Lin (right panels), and imaged by a fluorescent reader. All the colonies that were grown over the triple selective plates contained GFP, thus substantiating plasmid acquisition by the recipient bacteria. Yellow arrows indicate the donor cells lacking GFP and blue arrows indicate the recipient cells expressing GFP. The experiments were repeated at least 3 times independently with similar results.

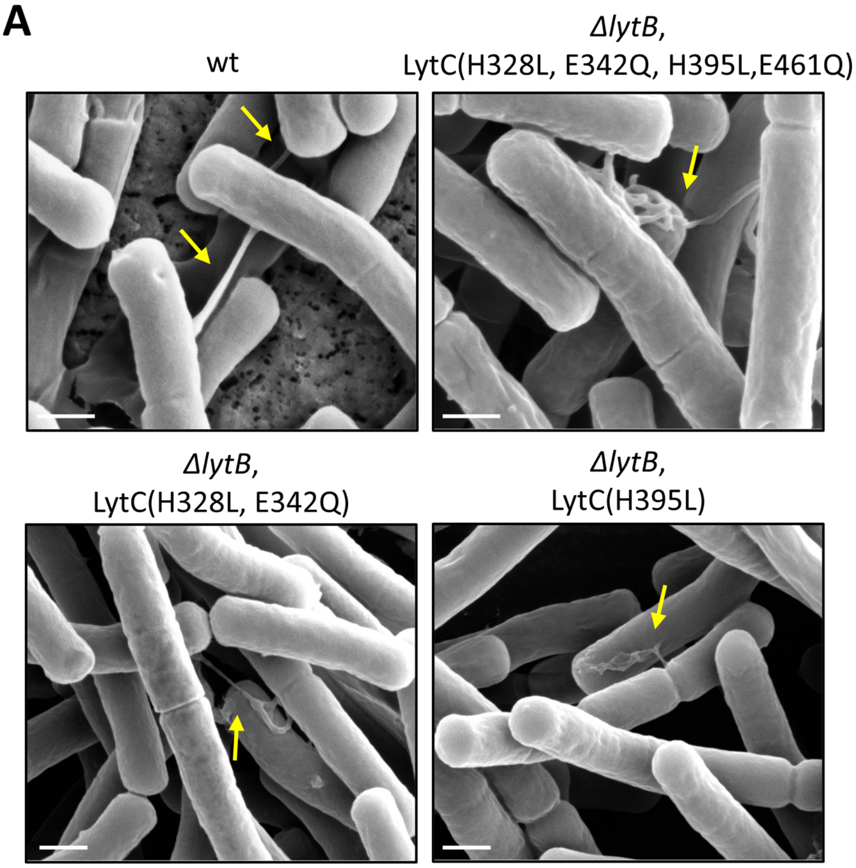
A
wt
ΔlytB,
LytC(H328L, E342Q, H395L,E461Q)
ΔlytB,
LytC(H328L, E342Q)
ΔlytB,
LytC(H395L)

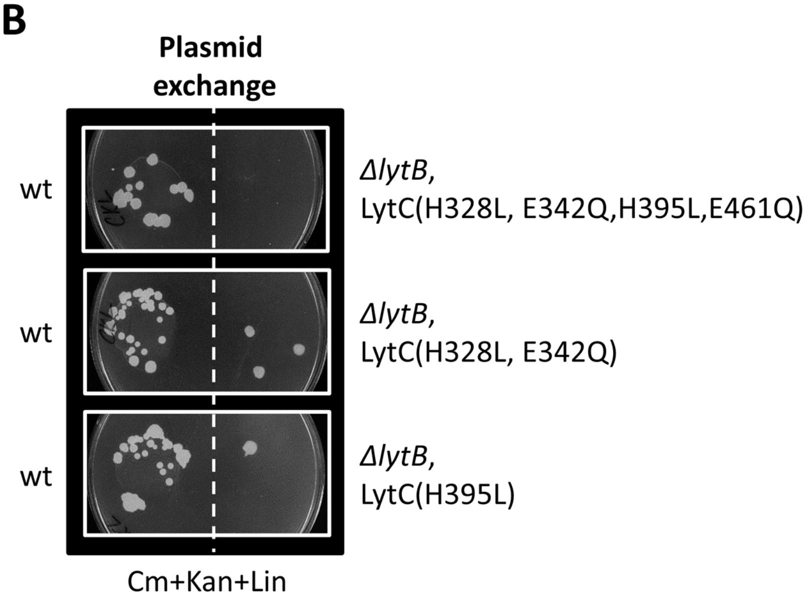
B
Plasmid
exchange
wt
wt
wt
ΔlytB,
LytC(H328L, E342Q,H395L,E461Q)
ΔlytB,
LytC(H328L, E342Q)
ΔlytB,
LytC(H395L)
Cm+Kan+Lin

**Supplementary Figure 3: LytC amidase activity affects nanotube formation**

(**A**) *Bs* wt (PY79) and strains deficient in LytC amidase activity [AB304 (Δ*lytB, lytC H328L-E342Q-H395L-E461Q*), AB305 (Δ*lytB, lytC H328L-E342Q*), AB306 (Δ*lytB, lytC H395L*)] (see Methods) were grown to mid logarithmic phase, spotted onto EM grids, incubated on LB agar plates for 4 h at 37°C, and visualized by XHR-SEM. Shown are typical field of cells with arrows indicating intercellular nanotube patterns. The point mutations in the LytC amidase activity decreased nanotube formation, showing the continual nanotube phenotype typical to that of Δ*lytBC*. Scale bar represents 500 nm. (**B**) Testing molecular exchange for lytC mutant strains. Pairs of donor (GD110: *amyE*::$P_{hyper\text{-}spank}$-*cat-spec*, pHB201/*cat, erm*) ($Cm^R$, $Spec^R$, $Mls^R$) and recipient (SB513: *amyE*::$P_{hyper\text{-}spank}$-*gfp-kan*) ($Kan^R$) parental strains (wt) were used. The investigated strains harbor the corresponding genotypes, with the indicated mutations in both donor and recipient strains. Donor and recipient cells were mixed in 1:1 ratio and incubated in LB supplemented with 1 mM IPTG for 4 h at 37°C with gentle shaking. Equal numbers of cells were then spotted onto and LB agar containing Cm, Kan and Lin. A sharp decrease in plasmid exchange was observed for *lytC* point mutant strains (right side of the plates), with the quadruple mutant exhibiting the most sever phenotype.

The experiments were repeated at least 3 times independently with similar results.

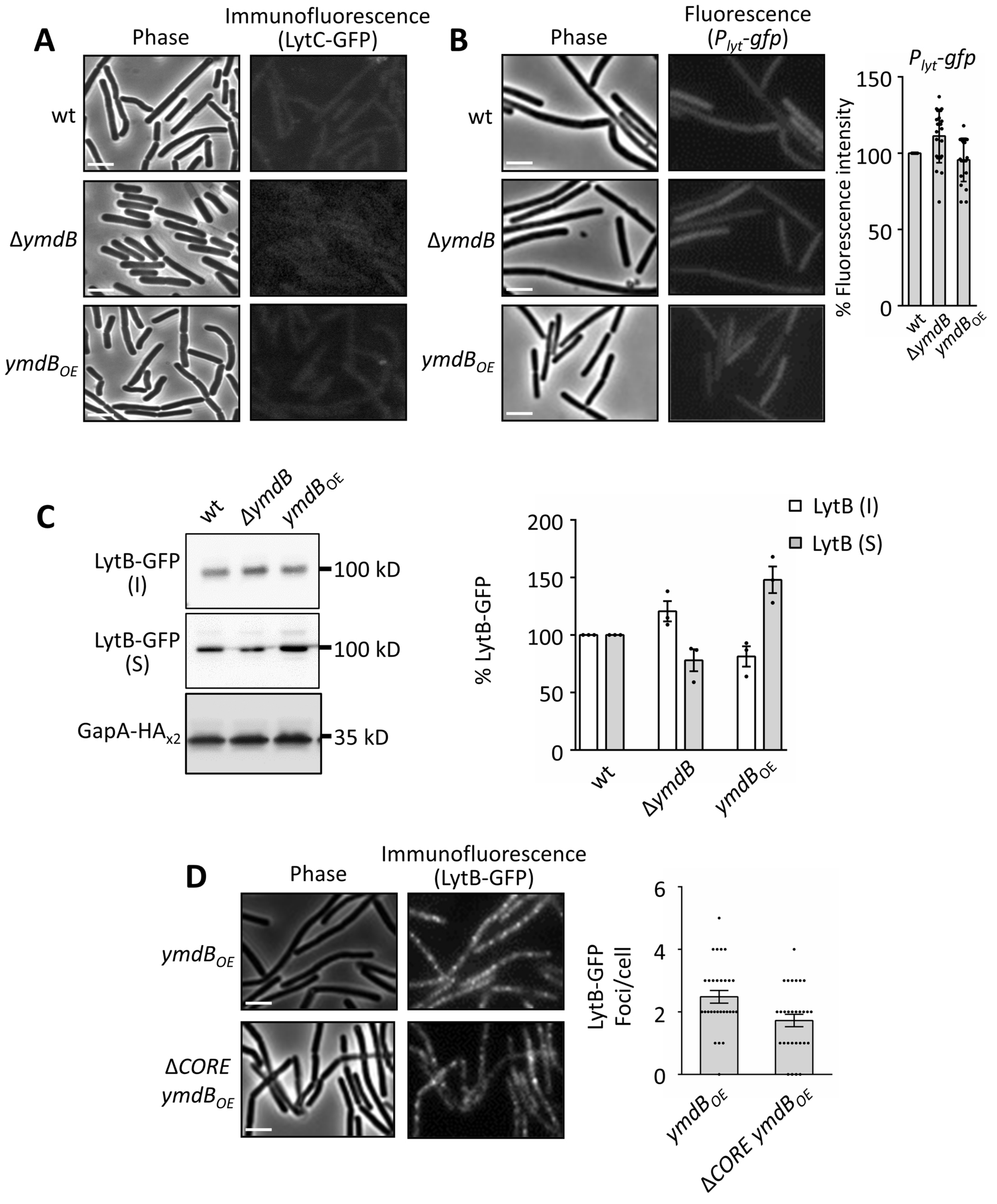
A
Phase
Immunofluorescence (LytC-GFP)
wt
ΔymdB
ymdB OE
B
Phase
Fluorescence (Plyt-gfp)
wt
ΔymdB
ymdB OE
Plyt-gfp
% Fluorescence intensity
150
100
50
0
wt
ΔymdB
ymdB OE
C
wt
ΔymdB
ymdB OE
LytB-GFP (I)
100 kD
LytB-GFP (S)
100 kD
GapA-HAx2
35 kD
LytB (I)
LytB (S)
% LytB-GFP
200
150
100
50
0
wt
ΔymdB
ymdB OE
D
Phase
Immunofluorescence (LytB-GFP)
ymdB OE
ΔCORE ymdB OE
LytB-GFP Foci/cell
6
4
2
0
ymdB OE
ΔCORE ymdB OE

**Supplementary Figure 4: Characterization of LytB and LytC localization**

(**A**) *Bs* wt (AB45: *lytC-gfp*), Δ*ymdB* (AB116: *lytC-gfp,* Δ*ymdB*) and $ymdB_{OE}$ (AB157: *lytB-gfp,* Δ*ymdB, amyE*::$P_{hyper\text{-}spank}$-*ymdB*) strains were spotted onto poly L-lysine coated coverslips, treated with anti-GFP primary antibodies and FITC-conjugated secondary antibodies and visualized by fluorescence microscopy. Shown are phase contrast images (left) and respective immunofluorescence images (right). The experiments were repeated at least 3 times independently with similar results.

(**B**) *Bs* wt (AB222: $P_{lytA}$-*gfp*), Δ*ymdB* (AB223a: $P_{lytA}$-*gfp,* Δ*ymdB*) and $ymdB_{OE}$ (AB223: $P_{lytA}$-*gfp,* Δ*ymdB, amyE*::$P_{hyper\text{-}spank}$-*ymdB*) strains containing *gfp* expressed from the promoter of *lytABC* operon were visualized by fluorescence microscopy. Shown are phase contrast images (left) and respective fluorescence images (right). Histogram presents quantitation of fluorescence signal obtained from the indicated strains. Shown are relative values (wt = 100%) of the GFP signal and mean ± SEM of at least 3 independent experiments ($n_{cells}$=500).

(**C**) *Bs* wt (AB278: *lytB-gfp*, *gapA*-$HA_{x2}$, Δ*hag*), Δ*ymdB* (AB280: *lytB-gfp*, *gapA*-$HA_{x2}$, Δ*ymdB*, Δ*hag*) and $ymdB_{OE}$ (AB279: *lytB-gfp*, *gapA*-$HA_{x2}$, Δ*ymdB*, *amyE*::$P_{hyper\text{-}spank}$-*ymdB*, Δ*hag*) strains were subjected to Western blot analysis. Initially, surface proteins were isolated using 1.5 M LiCl from equal number of cells from each strain, followed by intracellular protein isolation by treating the cells with lysozyme and lysis buffer. Shown is a Western blot analysis utilizing anti-GFP antibodies to indicate the levels of intracellular (I) and surface (S) LytB. Intracellular GapA-$HA_{x2}$ levels, detected on a separate western blot with anti-HA antibodies, serve as a loading control. Histogram presents quantification of LytB-GFP signal obtained from Western blot analysis

(arbitrary units). Shown are relative values (wt = 100%) and mean ± SEM of at least 3 independent experiments.

(**D**) *Bs ymdB*$_{OE}$ (AB144: *lytB-gfp, ΔymdB, amyE*::P$_{hyper\text{-}spank}$-*ymdB*) and Δ*CORE ymdB*$_{OE}$ (AB273: *lytB-gfp, ΔCORE, ΔymdB, amyE*::P$_{hyper\text{-}spank}$-*ymdB, Δhag*) strains were spotted onto poly L-lysine coated coverslips, treated with anti-GFP primary antibodies and FITC-conjugated secondary antibodies and visualized by fluorescence microscopy. Shown are phase contrast images (left) and respective immunofluorescence images (right). Graph presents quantification of the number of LytB-GFP foci displayed per cell. Shown are mean ± SEM of at least 3 independent experiments ($n_{cells}$=50).

Source data are provided as a Source Data file.

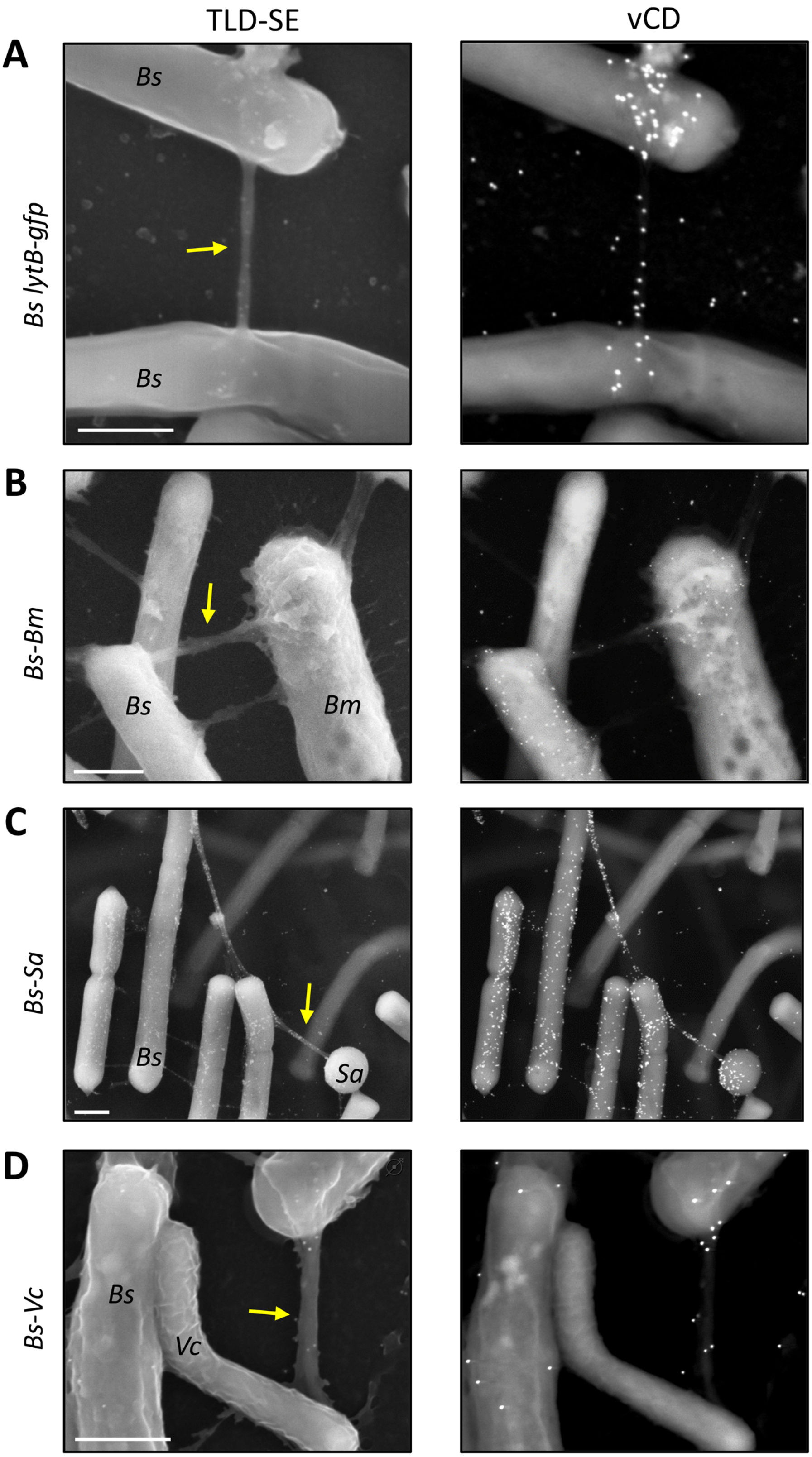
TLD-SE
vCD
A
Bs lytB-gfp
Bs
Bs
B
Bs-Bm
Bs
Bm
C
Bs-Sa
Bs
Sa
D
Bs-Vc
Bs
Vc

**Supplementary Figure 5: LytB molecules localized to intercellular nanotubes and reach the recipient cell surface**

(**A**) *Bs* cells expressing LytB-GFP (AB144: *lytB-gfp,* Δ*ymdB, amyE*::$P_{hyper\text{-}spank}$-*ymdB,* Δ*hag*) were spotted onto EM grids and subjected to immuno-gold XHR-SEM analysis, using primary antibodies against GFP and secondary gold-conjugated antibodies. Samples were not coated before observation. Shown is an XHR-SEM image acquired using TLD-SE (through lens detector-secondary electron) for nanotube visualization (left panel), and the corresponding vCD (low-kV high-contrast detector) for gold particle (white dots) detection (right panel).

(**B**) *Bs* cells expressing LytB-GFP (AB170: *lytB-gfp, ΔymdB, amyE*::$P_{hyper\text{-}spank}$-*ymdB,* Δ*hag,* Δ*wapA*) were mixed with *Bm* (OS2, lacking GFP), spotted onto EM grids and subjected to immuno-gold XHR-SEM as described in A. Shown is an XHR-SEM image acquired using TLD-SE for nanotube visualization (left panel), and the corresponding vCD for gold particle (white dots) detection (right panel).

(**C**) *Bs* cells expressing GFP tagged LytB (AB144: *lytB-gfp,* Δ*ymdB, amyE*::$P_{hyper\text{-}spank}$-*ymdB,* Δ*hag*) were mixed with *Sa* (MRSA, lacking GFP), spotted onto EM grids and subjected to immuno-gold XHR-SEM as described in A. Shown is an XHR-SEM image acquired using TLD-SE for nanotube visualization (left panel), and the corresponding vCD for gold particle (white dots) detection (right panel).

(**D**) *Bs* cells expressing GFP tagged LytB (AB144: *lytB-gfp,* Δ*ymdB, amyE*::$P_{hyper\text{-}spank}$-*ymdB,* Δ*hag*) were mixed with *Vc* (N16961, lacking GFP), spotted onto EM grids and subjected to immuno-gold XHR-SEM analysis as described in A. Shown is an XHR-SEM image acquired using TLD-SE for nanotube visualization (left panel), and the corresponding vCD for gold particle (white dots) detection (right panel).

Scale bars represent 500 nm. Arrows indicate the intercellular nanotubes.

This figure supplements the full original fields and modes used to capture the images presented in Figure 3.

The experiments were repeated at least 3 times independently with similar results.

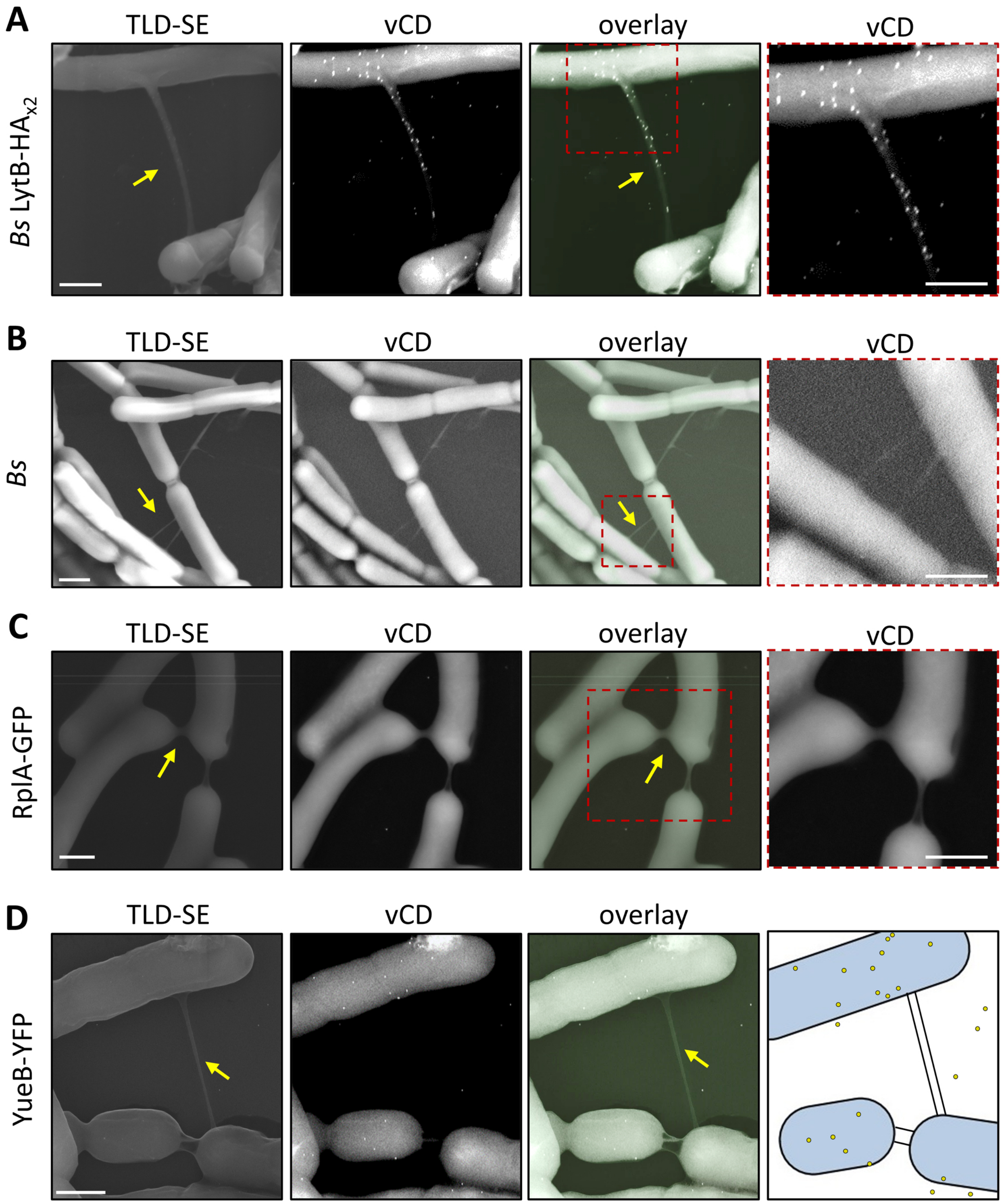

A
B
C
D
TLD-SE
vCD
overlay
Bs LytB-HA$_{x2}$
Bs
RplA-GFP
YueB-YFP

**Supplementary Figure 6: Immuno XHR-SEM analysis of various *Bs* proteins**

(**A**) *Bs* cells expressing LytB-$HA_{x2}$ (AB267: *lytB-$HA_{x2}$, ΔymdB, amyE*::$P_{hyper\text{-}spank}$-*ymdB, Δhag*) were spotted onto EM grids and subjected to immuno-gold XHR-SEM using primary antibodies against HA and secondary gold-conjugated antibodies. Samples were not coated before observation. Shown are (left to right): an XHR-SEM image acquired using TLD-SE for nanotube visualization, a corresponding vCD for gold particle (white dots) detection, an overlay of XHR-SEM images acquired using TLD-SE and vCD, and a corresponding vCD image of the boxed region in the overlay image.

(**B**) *Bs* cells lacking GFP (GB168: *ΔymdB, amyE*::$P_{hyper\text{-}spank}$-*ymdB, Δhag*) were spotted onto EM grids and subjected to immuno-gold XHR-SEM using primary antibodies against GFP and secondary gold-conjugated antibodies. Samples were not coated before observation. Shown are (left to right): an XHR-SEM image acquired using TLD-SE for nanotube visualization, a corresponding vCD for gold particle detection (white dots, not detected), an overlay of XHR-SEM images acquired using TLD-SE and vCD, and a corresponding vCD image of the boxed region in the overlay image.

(**C**) *Bs* cells expressing RplA-GFP (AR5: *rplA-gfp*) were spotted onto EM grids and subjected to immuno-gold XHR-SEM as described in B. Samples were not coated before observation. Shown are (left to right): an XHR-SEM image acquired using TLD-SE for nanotube visualization, a corresponding vCD for gold particle detection (white dots, not detected), an overlay of XHR-SEM images acquired using TLD-SE and vCD, and a corresponding vCD image of the boxed region in the overlay image.

(**D**) *Bs* cells expressing YueB-YFP (ET13: *amyE*::$P_{hyper\text{-}spank}$-*yueB-yfp*) were spotted onto EM grids and subjected to immuno-gold XHR-SEM as described in B. Samples were not coated before observation. Shown are (left to right): an XHR-SEM image acquired

using TLD-SE for nanotube visualization, a corresponding vCD for gold particle (white dots) detection, an overlay of XHR-SEM images acquired using TLD-SE and vCD, and a schematic that depicts the interpretive cell layout, highlighting the region with gold signal captured by XHR-SEM.

The experiments were repeated at least 3 times independently with similar results. Scale bars represent 500 nm. Arrows indicate the intercellular nanotubes.

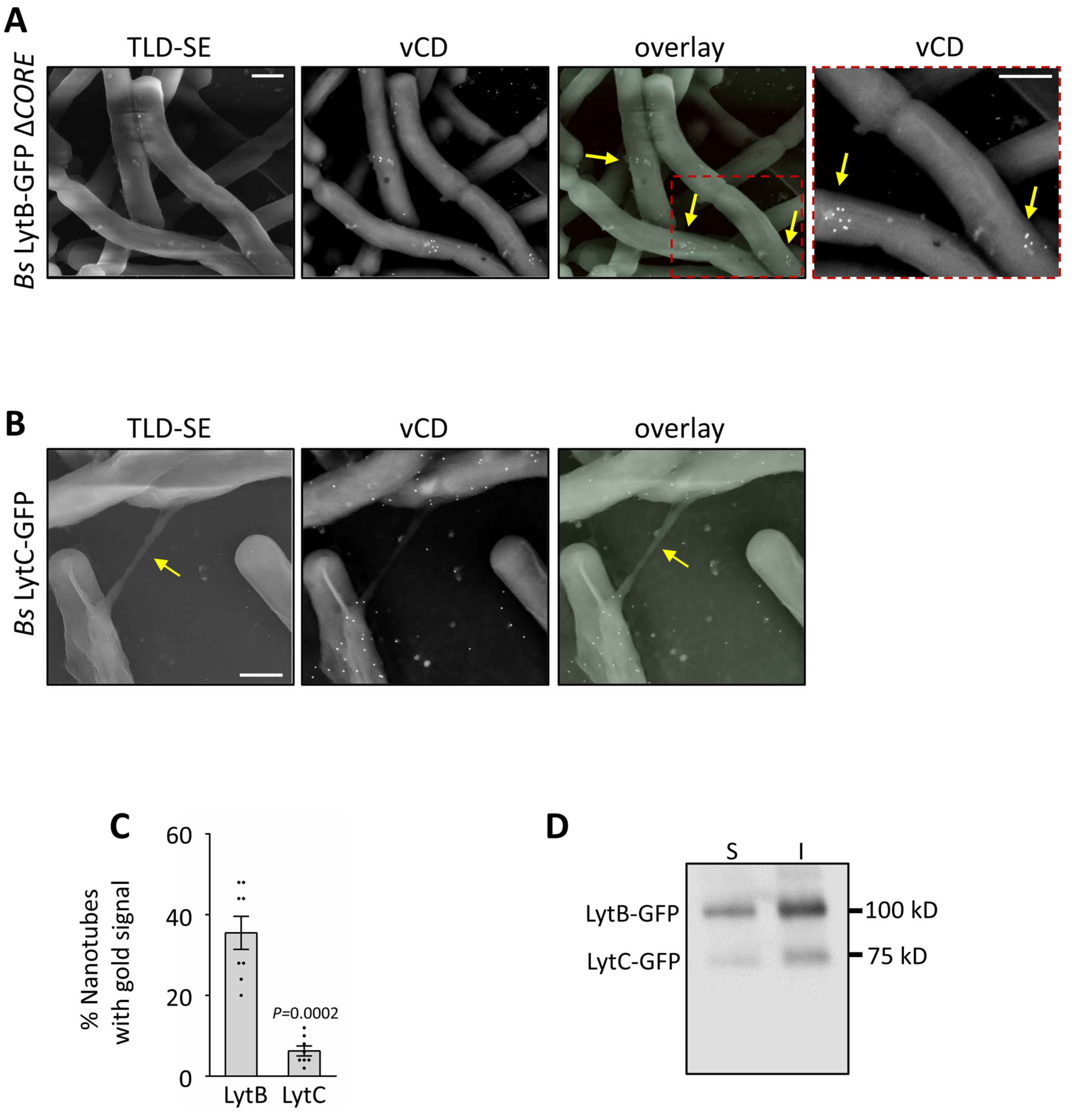
A
Bs LytB-GFP ΔCORE
TLD-SE
vCD
overlay
vCD
B
Bs LytC-GFP
TLD-SE
vCD
overlay
C
% Nanotubes with gold signal
60
40
20
0
LytB
LytC
P=0.0002
D
S
I
LytB-GFP
LytC-GFP
100 kD
75 kD

**Supplementary Figure 7**: **Immuno XHR-SEM analysis of LytB and LytC localization**

(**A**) *Bs* Δ*CORE* cells expressing LytB-GFP (AB273: *lytB-gfp, ΔCORE, ΔymdB, amyE*::$P_{hyper\text{-}spank}$-*ymdB, Δhag*) were spotted onto EM grids and subjected to immuno-gold XHR-SEM using primary antibodies against GFP and secondary gold-conjugated antibodies. Shown are (left to right): an XHR-SEM image acquired using TLD-SE for nanotube visualization, a corresponding vCD for gold particle (white dots) detection, an overlay of XHR-SEM images acquired using TLD-SE and vCD, and a corresponding vCD image of the boxed region in the overlay image. Arrows indicate the LytB foci. The experiment was repeated at least 3 times independently with similar results.

(**B**) *Bs* cells expressing LytC-GFP (AB157: *lytC-gfp, ΔymdB, amyE*::$P_{hyper\text{-}spank}$-*ymdB, Δhag*) were spotted onto EM grids and subjected to immuno-gold XHR-SEM as described in A. Shown are (left to right): an XHR-SEM image acquired using TLD-SE for nanotube visualization, a corresponding vCD for gold particle (white dots) detection, and an overlay of XHR-SEM images acquired using TLD-SE and vCD modes. Arrow indicates the intercellular nanotube.

(**C**) Quantitation of number of nanotubes containing gold signal from strains expressing LytB-GFP (AB144: *lytB-gfp, ΔymdB, amyE*::$P_{hyper\text{-}spank}$-*ymdB, Δhag*) or LytC-GFP (AB157: *lytC-gfp, ΔymdB, amyE*::$P_{hyper\text{-}spank}$-*ymdB, Δhag*) following immuno-gold XHR-SEM analysis as described in A. Shown are mean ± SEM and *P* values (unpaired student's t-test) of at least 3 independent experiments ($n_{nanotubes}$=100).

(**D**) *Bs* strain containing both LytB and LytC tagged with GFP (AB287: *lytB-gfp*, *lytC-gfp*) was subjected to Western blot analysis. Initially, surface proteins (S) were isolated using 1.5 M LiCl, followed by intracellular protein (I) isolation by treating the cells with

lysozyme and lysis buffer. Shown is a Western blot analysis utilizing anti-GFP antibodies to indicate the levels of both LytB and LytC. The experiment was repeated at least 3 times independently with similar results.

Scale bars represent 500 nm.

Source data are provided as a Source Data file.

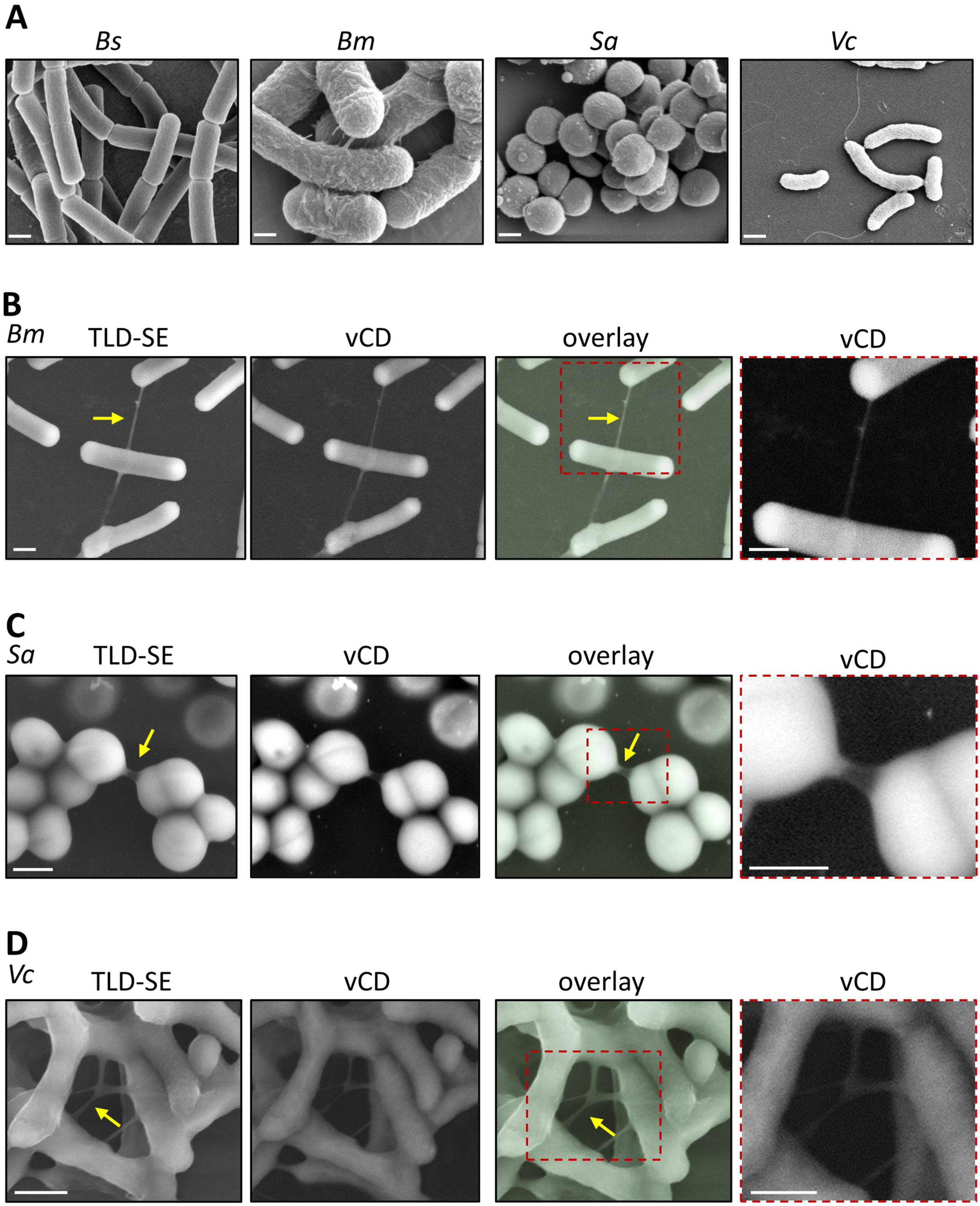
A
Bs
Bm
Sa
Vc
B
Bm
TLD-SE
vCD
overlay
vCD
C
Sa
TLD-SE
vCD
overlay
vCD
D
Vc
TLD-SE
vCD
overlay
vCD

**Supplementary Figure 8: XHR-SEM analysis of various bacterial species**

(**A**) *Bs* (PY79), *Bm* (OS2), *Sa* (MRSA) and *Vc* (N16961) strains were grown to mid logarithmic phase, spotted onto poly L-lysine coated coverslips and visualized by XHR-SEM.

(**B-D**) *Bm* (OS2) (**B**), *Sa* (MRSA) (**C**) or *Vc* (N16961) (**D**) strains, lacking GFP, were spotted onto EM grids and subjected to immuno-gold XHR-SEM using primary antibodies against GFP and secondary gold-conjugated antibodies. Samples were not coated before observation. Shown are (left to right): an XHR-SEM image acquired using TLD-SE for nanotube visualization, a corresponding vCD for gold particle detection (white dots, not detected), an overlay of XHR-SEM images acquired using TLD-SE and vCD, and a corresponding vCD image of the boxed region in the overlay image. Arrows indicate the intercellular nanotubes.

The experiments were repeated at least 3 times independently with similar results.

Scale bars represent 500 nm.

**A**

**B**

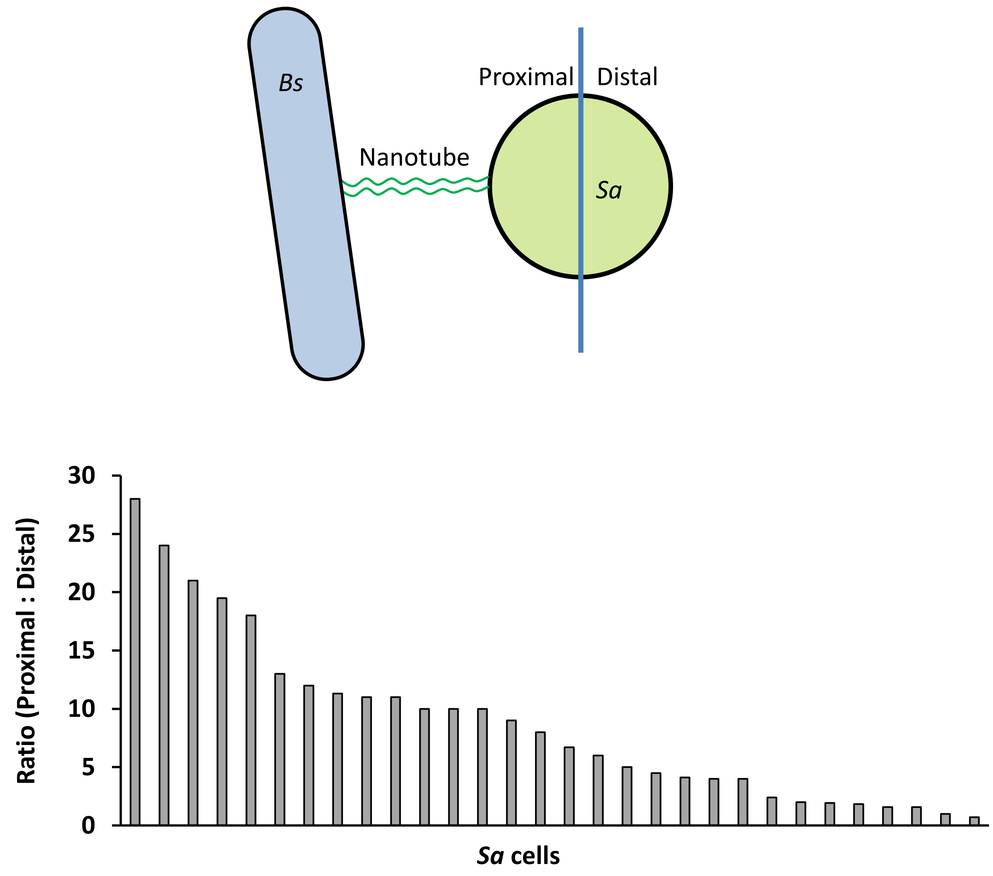


**Supplementary Figure 9: Quantitation analysis of LytB molecules on *Sa* surface**

(**A**) Schematic depicting the *Sa* cells (green) divided into proximal half containing the nanotube attachment site and a distal half opposing the nanotube attachment site. (**B**) Histogram showing the ratio between the number of gold particles detected between the proximal and distal halves of different *Sa* cells (n=30).

Source data are provided as a Source Data file.

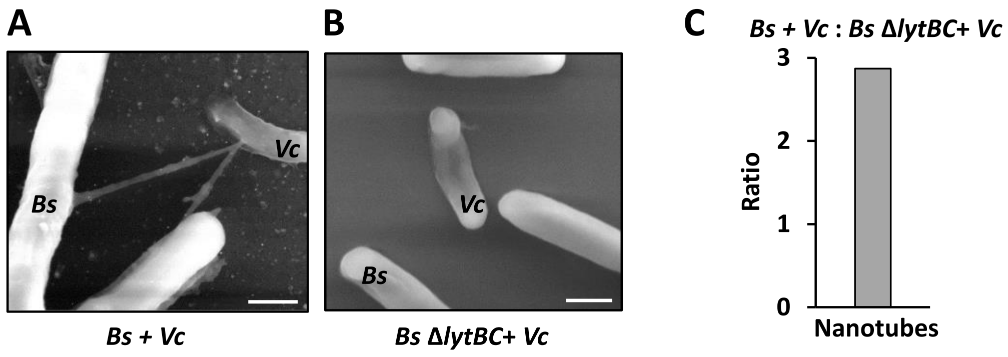


**Supplementary Figure 10: Nanotube formation between *Bs* and *Vc* cells**

(**A**-**B**) *Bs* (GD215: Δ*hag*) and *Vc* wt strains (**A**) or *Bs* Δ*lytBC* (AB64: Δ*lytBC*, Δ*hag*) and *Vc* wt strains (**B**) were mixed, spotted onto EM grids, incubated on LB agar plates for 4 h at 37°C, and visualized by XHR-SEM. Scale bars represent 500 nm. (**C**) Histogram showing the ratio between the number of interspecies nanotubes detected in A and B ($n_{cells}$=500). The experiment was repeated at least 3 times independently with similar results.

Source data are provided as a Source Data file.

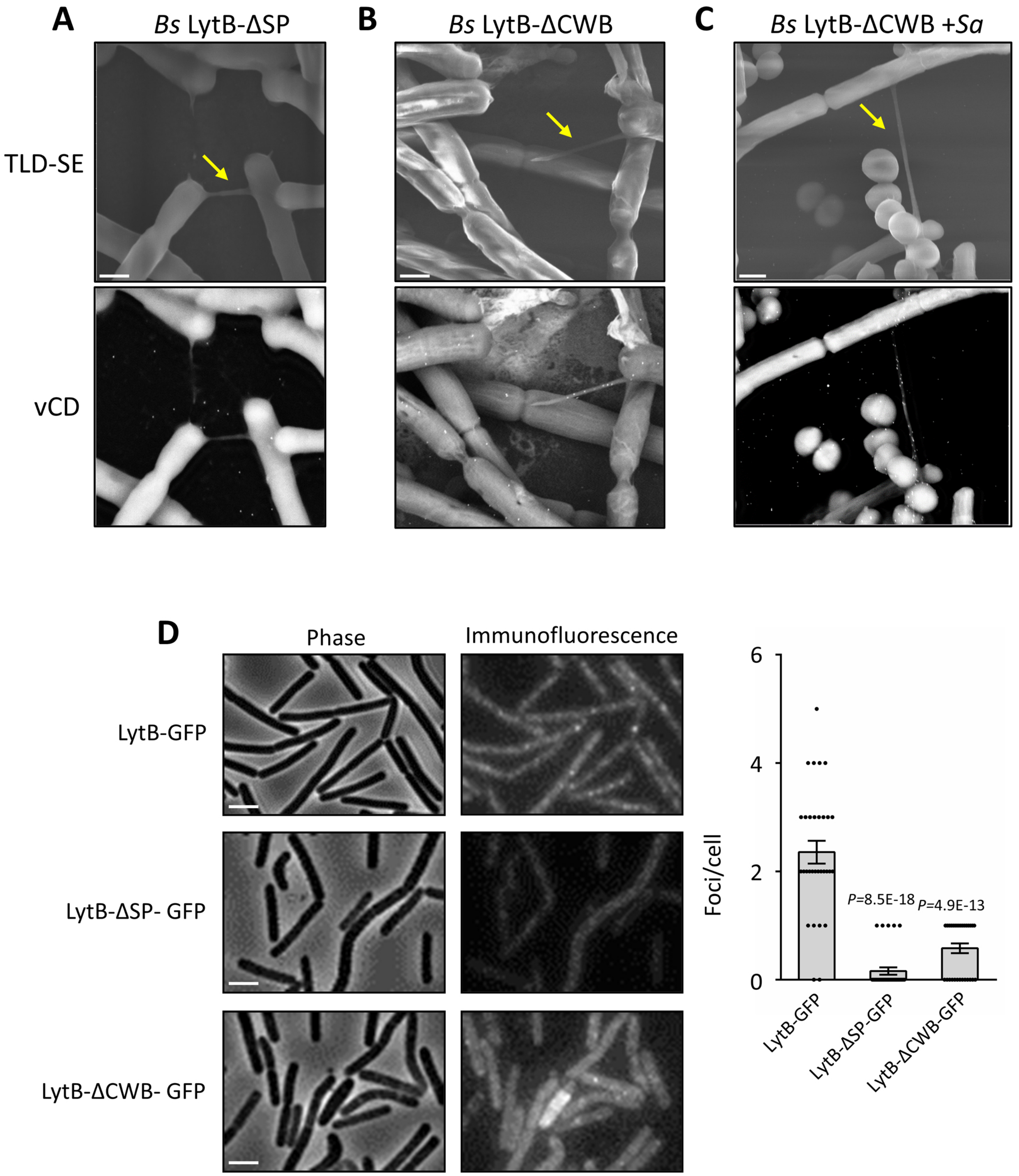

A
Bs LytB-ΔSP
B
Bs LytB-ΔCWB
C
Bs LytB-ΔCWB +Sa
TLD-SE
vCD
D
Phase
Immunofluorescence
LytB-GFP
LytB-ΔSP- GFP
LytB-ΔCWB- GFP
Foci/cell
6
4
2
0
P=8.5E-18
P=4.9E-13
LytB-GFP
LytB-ΔSP-GFP
LytB-ΔCWB-GFP

**Supplementary Figure 11: Analysis of LytB domains**

(**A-B**) *Bs* cells expressing GFP tagged LytB-ΔSP (AB263: *lytB-ΔSP-gfp*, Δ*ymdB*, *amyE*::P*hyper-spank*-*ymdB*, Δ*hag*) (**A**) or GFP tagged LytB-ΔCWB (AB250: *lytB-ΔCWB-gfp, amyE*::P*hyper-spank*-*ymdB,* Δ*hag*) (**B**) were spotted onto EM grids and subjected to immuno-gold XHR-SEM analysis, using primary antibodies against GFP and secondary gold-conjugated antibodies. Samples were not coated before observation. Shown is an XHR-SEM image acquired using TLD-SE for nanotube visualization, and the corresponding vCD for gold particle (white dots) detection. Arrows indicate nanotubes. The experiments were repeated at least 3 times independently with similar results. Scale bar represents 500 nm.

(**C**) *Bs* cells expressing GFP tagged LytB-ΔCWB (AB250) were mixed with *Sa* (MRSA, lacking GFP), spotted onto EM grids and subjected to immuno-gold XHR-SEM as described in A-B. Shown is an XHR-SEM image acquired using TLD-SE for nanotube visualization, and the corresponding vCD for gold particle (white dots) detection. Arrow indicates a nanotube. The experiment was repeated at least 3 times independently with similar results. Scale bar represents 500 nm.

(**D**) *Bs* strains containing GFP tagged LytB (AB144: *lytB-gfp,* Δ*ymdB, amyE*::P*hyper-spank*-*ymdB,* Δ*hag*), LytB-ΔSP (AB263: *lytB-ΔSP-gfp*, Δ*ymdB*, *amyE*::P*hyper-spank*-*ymdB*, Δ*hag*) or LytB-ΔCWB (AB250: *lytB-ΔCWB-gfp, amyE*::P*hyper-spank*-*ymdB,* Δ*hag*) were spotted onto poly L-lysine coated coverslips, treated with anti-GFP primary antibodies and FITC-conjugated secondary antibodies and visualized by fluorescence microscopy. Shown are phase contrast images (left) and respective immunofluorescence images (right). Histogram presents the quantitation of the number of LytB-GFP foci displayed per cell.

Shown are mean ± SEM and *P* values (unpaired student's t-test) of at least 3 independent experiments ($n_{cells}$=200). Scale bars represent 5 µm.

A-C panels supplement the full original fields and modes used to capture the images presented in Figure 4.

Source data are provided as a Source Data file.

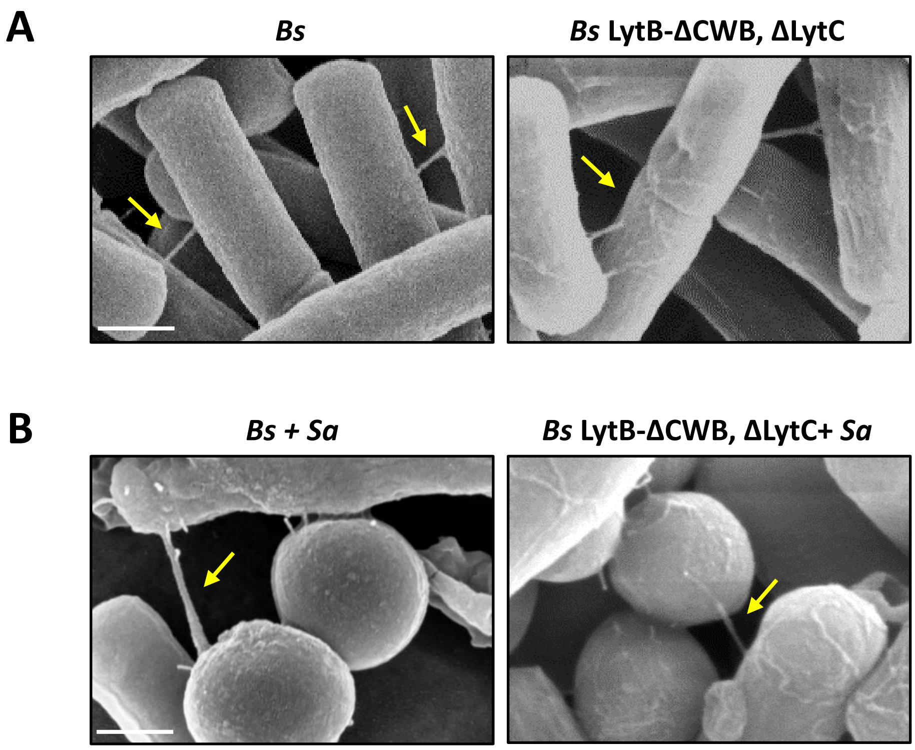


**Supplementary Figure 12: LytB CWB domains are required for nanotube penetration**

(**A**) *Bs* wt (PY79) or LytB-ΔCWB, ΔLytC (AB286: *lytB-ΔCWB, ΔlytC*) strains were grown to the mid logarithmic phase, spotted onto EM grids, incubated on LB agar plates for 4 h at 37°C, and visualized by XHR-SEM.

(**B**) *Bs* wt (PY79) or LytB-ΔCWB, ΔLytC (AB286: *lytB-ΔCWB*, *ΔlytC*) strains were grown to mid logarithmic phase, mixed with mid-log *Sa* (MRSA) cells, spotted onto EM grids, incubated on LB agar plates for 4 h at 37°C, and visualized by XHR-SEM.

The experiments were repeated at least 3 times independently with similar results.

Arrows indicate intercellular nanotubes. Scale bars represent 500 nm.

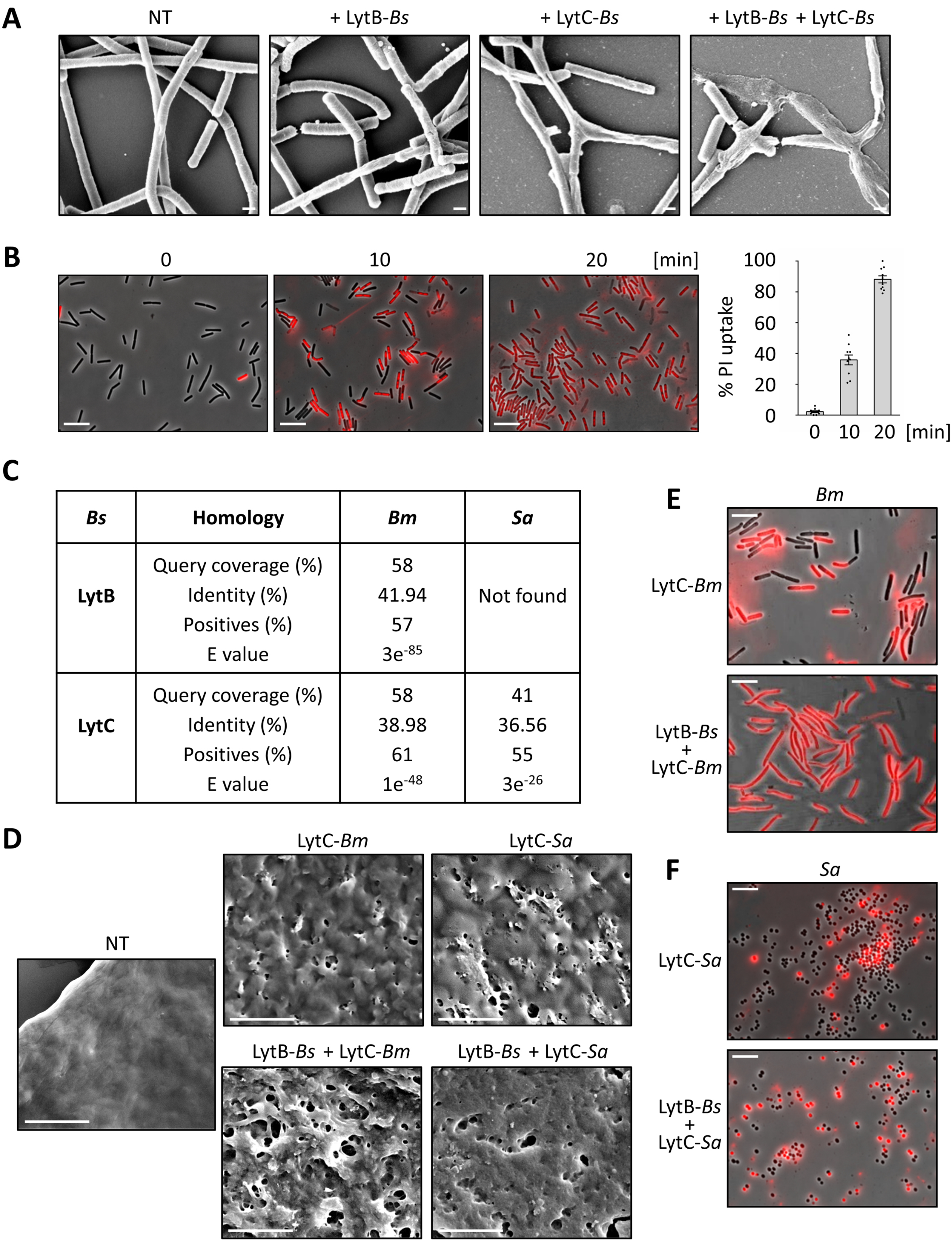


| ***Bs*** | **Homology** | ***Bm*** | ***Sa*** |
|---|---|---|---|
| **LytB** | Query coverage (%) | 58 | Not found |
| | Identity (%) | 41.94 | |
| | Positives (%) | 57 | |
| | E value | $3e^{-85}$ | |
| **LytC** | Query coverage (%) | 58 | 41 |
| | Identity (%) | 38.98 | 36.56 |
| | Positives (%) | 61 | 55 |
| | E value | $1e^{-48}$ | $3e^{-26}$ |

**Supplementary Figure 13: Visualizing LytB and LytC exogenous activity on *Bs* CW**

(**A**) *Bs* wt (PY79) cells were incubated for 30 min with purified LytB-*Bs*, LytC-*Bs* or both, spotted onto poly L-lysine coated coverslips and visualized by XHR-SEM. Scale bar represents 500 nm.

(**B**) *Bs* wt (PY79) cells were incubated with 10 µM of lysozyme at the indicated time points, stained with PI and visualized by fluorescence microscopy. Shown are overlay images of phase contrast (grey) and fluorescence from PI staining (red). Scale bar represent 5 µm. Graph presents the PI uptake. Shown is the percentage of PI-labeled cells and mean ± SEM of at least 3 independent experiments ($n_{cells}$=500).

(**C**) Homologs of LytB-*Bs* and LytC-*Bs* were identified in the genomes of *Bm* and *Sa* using NCBI and KEGG genome databases. Shown are the percent homology (%) between LytB-*Bs* and LytC-*Bs* and their homologues.

(**D**) *Bs* CW was incubated for 30 min with purified LytC-*Bm* with or without LytB-*Bs*, or with purified LytC-*Sa* with or without LytB-*Bs*, spotted onto poly L-lysine coated coverslips and visualized by XHR-SEM. Scale bars represent 500 nm.

(**E**) *Bm* (OS2) cells were incubated for 10 min with purified LytC-*Bm* with or without LytB-*Bs*, stained with PI and visualized by fluorescence microscopy. Shown are overlay images of phase contrast (grey) and fluorescence from PI staining (red). Scale bars represent 5 µm.

(**F**) *Sa* (MRSA) cells were incubated for 10 min with purified LytC-*Sa* with or without LytB-*Bs* (**b**), stained with PI and visualized by fluorescence microscopy. Shown are overlay images of phase contrast (grey) and fluorescence from PI staining (red). Scale bars represent 5 µm.

Experiments were repeated at least 3 times independently with similar results.

Source data are provided as a Source Data file.

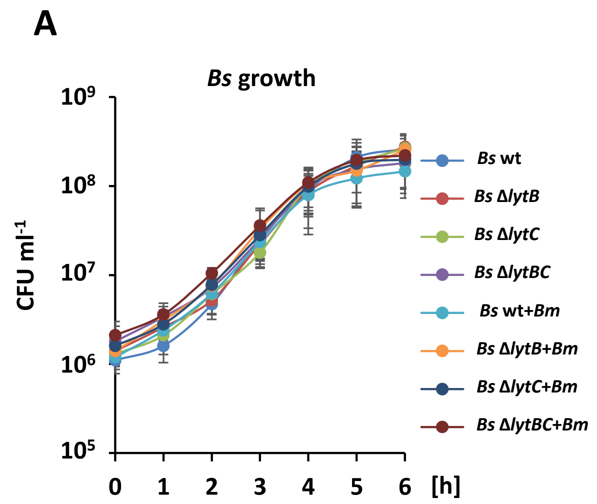


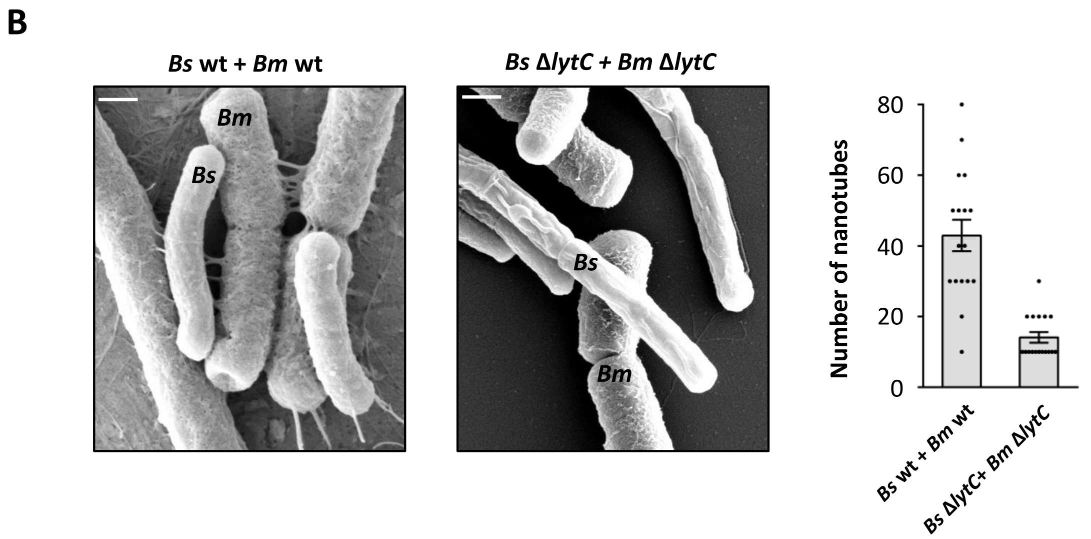


**Supplementary Figure 14: Compatibility between CW lytic proteins determines interspecies nanotube formation**

(**A**) The indicated *Bs* strains were grown separately or in a mixture with the indicated *Bm* strains in LB medium at 37°C. Cells were plated for CFUs at the indicated time points. Each point represents an average value and SD of three independent experiments.

(**B**) *Bs* (GD215: Δ*hag*) and *Bm* (OS2) wt strains or *Bs* ΔLytC (AB62: *ΔlytC*, Δ*hag*) and *Bm* Δ*lytC* (ABm2) strains were mixed, spotted onto EM grids, incubated on LB agar plates

for 4 h at 37°C, and visualized by XHR-SEM. Scale bars represent 500 nm. Graph shows mean ± SEM of the number of nanotubes displayed per 50 interspecies pairs detected between the indicated strains ($n_{cell\ pairs}$=200).

Source data are provided as a Source Data file.

## References (related to Supplementary Data 1)


1 Youngman, P., Perkins, J. B. & Losick, R. Construction of a cloning site near one end of Tn917 into which foreign DNA may be inserted without affecting transposition in *Bacillus subtilis* or expression of the transposon-borne erm gene. *Plasmid* **12**, 1-9 (1984).

2 Dubey, G. P. & Ben-Yehuda, S. Intercellular nanotubes mediate bacterial communication. *Cell* **144**, 590-600 (2011).

3 Rosenberg, A., Sinai, L., Smith, Y. & Ben-Yehuda, S. Dynamic expression of the translational machinery during Bacillus subtilis life cycle at a single cell level. *PloS One* **7**, e41921 (2012).

4 Tzipilevich, E., Habusha, M. & Ben-Yehuda, S. Acquisition of Phage Sensitivity by Bacteria through Exchange of Phage Receptors. *Cell* **168**, 186-199 (2017).

5 Dubey, G. P. *et al.* Architecture and characteristics of bacterial nanotubes. *Dev Cell* **36**, 453-461 (2016).

6 Stempler, O. *et al.* Interspecies nutrient extraction and toxin delivery between bacteria. *Nat Commun* **8**, 315 (2017).

7 Bron, S. *et al.* Protein secretion and possible roles for multiple signal peptidases for precursor processing in bacilli. *Journal of Biotechnology* **64**, 3-13 (1998).

8 Lemon, K. P. & Grossman, A. D. Localization of bacterial DNA polymerase: evidence for a factory model of replication. *Science* **282**, 1516-1519 (1998).

9 Guerout-Fleury, A. M., Frandsen, N. & Stragier, P. Plasmids for ectopic integration in Bacillus subtilis. *Gene* **180**, 57-61 (1996).
