# Supplementary material for: Donor-delivered cell wall hydrolases facilitate nanotube penetration into recipient bacteria
Source: Nat Commun. 2020 Apr 22;11:1938. doi: 10.1038/s41467-020-15605-1 (PMC7176660; doi:10.1038/s41467-020-15605-1)
Supplement: Supplementary file 3 — Description of Additional Supplementary Files [file 41467_2020_15605_MOESM3_ESM.pdf]

Description of Additional Supplementary Files

File Name: Supplementary Data 1

Description: List of strains and plasmids used in this study

File Name: Supplementary Data 2

Description: List of primers used in this study
